# Daily *Plasmodium yoelii *infective mosquito bites do not generate protection or suppress previous immunity against the liver stage

**DOI:** 10.1186/1475-2875-10-97

**Published:** 2011-04-18

**Authors:** Tzvi Pollock, Ricardo Leitao, Cristina Galan-Rodriguez, Kurt A Wong, Ana Rodriguez

**Affiliations:** 1 Department of Microbiology, Division of Medical Parasitology, New York University School of Medicine, New York, NY 10010, USA; 2 Current Address: The Scripps Research Institute, La Jolla, CA 92037, USA

## Abstract

**Background:**

Human populations that are naturally subjected to *Plasmodium *infection do not acquire complete protection against the liver stage of this parasite despite prolonged and frequent exposure. However, sterile immunity against *Plasmodium *liver stage can be achieved after repeated exposure to radiation attenuated sporozoites. The reasons for this different response remain largely unknown, but a suppressive effect of blood stage *Plasmodium *infection has been proposed as a cause for the lack of liver stage protection.

**Methods:**

Using *Plasmodium yoelii *17XNL, the response generated in mice subjected to daily infective bites from normal or irradiated mosquitoes was compared. The effect of daily-infected mosquito bites on mice that were previously immunized against *P. yoelii *liver stage was also studied.

**Results:**

It was observed that while the bites of normal infected mosquitoes do not generate strong antibody responses and protection, the bites of irradiated mosquitoes result in high levels of anti-sporozoite antibodies and protection against liver stage *Plasmodium *infection. Exposure to daily infected mosquito bites did not eliminate the protection acquired previously with a experimental liver stage vaccine.

**Conclusions:**

Liver stage immunity generated by irradiated versus normal *P. yoelii *infected mosquitoes is essentially different, probably because of the blood stage infection that follows normal mosquito bites, but not irradiated. While infective mosquito bites do not induce a protective liver stage response, they also do not interfere with previously acquired liver stage protective responses, even if they induce a complete blood stage infection. Considering that the recently generated anti-malaria vaccines induce only partial protection against infection, it is encouraging that, at least in mouse models, immunity is not negatively affected by subsequent exposure and infection with the parasite.

## Background

Malaria is a widespread mosquito-borne infectious disease that kills almost one million persons per year, most of which are children. Growing scientific knowledge and the recent malaria research trend provide unparalleled opportunities to develop a malaria vaccine. Although the subunit vaccines are the most advanced in research pipelines and field clinical trials [1], an effective and focused effort prevails in order to develop an attenuated sporozoite-based vaccine [2,3], once the protective immunity produced by attenuated sporozoites is stronger and more unswerving than that induced by subunit vaccination [4]. Immunization with irradiated *Plasmodium *sporozoites grants protection against inoculation with non-attenuated sporozoites in mice models and humans [5,6]. However, individuals in malaria endemic regions do not develop fully protective immune responses against *Plasmodium *liver stage infections even if exposed to more than two infective mosquito bites daily [7,8], as is the case in high transmission areas [9]. Using a mouse model, a previous study showed that the mode of irradiated sporozoite delivery: daily mosquito bites (as in endemic areas) versus few large doses of sporozoites (as in immunization protocols) does not affect the generation of protective liver stage immunity [10]. In this work, it was assessed whether the daily bites of non-irradiated infected mosquitoes would also induce a protective liver stage immune response, similar to that induced by irradiated infected mosquito bites. This situation would be analogous to the circumstances of humans in high-transmission malaria endemic areas, where people are subjected to daily bites of infected mosquitoes [9].

In this work, it was observed that daily bites of non-irradiated infected mosquitoes do not induce liver stage protection, while the daily bites of irradiated mosquitoes do. This result again confirms the concordance of observations in mice and in humans regarding liver stage immunity and focuses the questions about generation of liver stage protection on the differences between infection generated by irradiated versus normal sporozoites.

Since the use of partially protective malaria liver stage vaccines in the field may soon be a reality [11], how would infected mosquito bites affect the immune response and protection induced by the vaccine becomes a highly relevant question. An enhancing effect caused by additional boosting liver stage antigens could be expected, however, an inhibition of the vaccine-induced protection has also been proposed [12]. Therefore, a mouse model was used to test whether the bites of infected mosquitoes could affect previously acquired liver stage immunity. Interestingly, it was found that after immunization of the mice, daily bites of infected mosquitoes do not significantly affect the liver stage protective response.

## Methods

### Parasites, mosquitoes and mouse immunizations

*Anopheles stephensii *mosquitoes were maintained as described [13] and infected with *P. yoelii *17XNL. This parasite line has been grown for over ten years without cloning and is the same used in previous work [10]. Irradiated mosquitoes were generated by exposure to 12 krad (120 Gy) of γ-irradiation (MDS Nordion Gammacell 1000 Elite). Female Swiss-Webster mice (NIH, Bethesda MD) were used for all experiments. For the partial immunization, each mouse was injected intravenously with 75,000 *P. yoelii *17XNL irradiated (12 krad) sporozoites dissected from the salivary glands of infected mosquitoes, followed two weeks later with a dose of 50,000. For daily mosquito bites, each mouse had two mosquitoes feed on the tail for 3 min. This was repeated daily for 6 weeks. Parasitaemia was followed regularly using thin blood smears from a drop of tail blood.

### Immunofluorescence titration of serum antibodies

For titration of *P. yoelii*-specific antibody levels, 10^4 ^salivary-gland sporozoites in each well were air-dried on glass multiwell IFA slides. Mouse serum was titrated and primary antibody bound to sporozoites was detected using FITC-labelled antimouse IgG (Sigma). A monoclonal *P. yoelii *CS-specific antibody (2F6) was used as a positive control (kindly provided by Dr. Photini Sinnis, NYU).

### Challenge and quantitative real-time PCR

Challenge of mice was performed in groups of five mice by i.v. injection of 10^4 ^(non-irradiated) *P. yoelii *17XNL GFP sporozoites [14], freshly dissected from mosquito salivary glands. Forty hours later, livers were harvested, total RNA was isolated, and malaria infection was quantified using reverse transcription followed by real-time PCR with primers that recognize GFP-specific sequences: 5'-GTC AGT GGA GAG GGT GAA GG-3' and 5'-ACT TCA GCA CGT GTC TTG TAG TTC-3'. Reaction was run for 40 cycles. Threshold cycle is the fractional cycle at which the fluorescence signal passes the threshold, which was set up at 50% of the maximal fluorescence signal.

### Statistical analysis

Data were analyzed using Prism (v. 4.0c, GraphPad). ANOVA test was performed as mentioned. Statistics were considered significant if *P *< 0.05.

## Results and discussion

**Figure 1:**
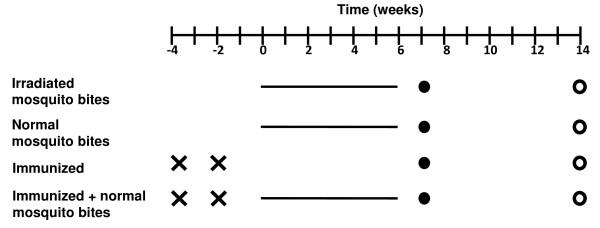
**Experiment schedule**. Four groups of five mice received different immunizations and were subjected to the bites of irradiated or normal infected mosquito bites as indicated. Two groups were immunized by intravenous injection of two doses of irradiated sporozoites two weeks apart (represented by crosses in the figure). After another two weeks, three of the four groups were subjected to two daily bites of infected mosquitoes, irradiated (dashed line) or not (continuous line) for six weeks. One week later (black circles), a small volume of serum was extracted from each mouse to determine anti-sporozoite antibody levels (results shown in Figure 3). Eight weeks later mice were challenged (white circles) and liver stage infection detected with real-time PCR (results shown in Table 1).

To compare the responses of mice to daily normal or irradiated mosquito bites, a protocol previously shown to induce protection in mice when mosquitoes infected with the non-lethal parasite *P. yoelii *17XNL are irradiated was followed [10]. The first group received an immunization regimen of daily bites from two irradiated mosquitoes to mimic an entomological inoculation rate found in regions of very high transmission [9,15]. The second group received identical treatment, but mice were bitten by non-irradiated infected mosquitoes. For both groups, each mouse had two mosquitoes feed on her tail for three minutes. This was repeated daily for six weeks (Figure 1).

To study whether the immune response induced by a vaccine can be affected by subsequent bites of infected mosquitoes, we immunized mice with two doses of radiation-attenuated sporozoites. This immunization protocol that induces only partial liver stage protection was purposely chosen to reflect the situation with current human liver stage vaccine prototypes [11]. After immunization, mice were subjected or not to daily bites of infected mosquitoes, as described above (Figure 1).

One week after the last mosquito bite a small volume of serum was obtained from each mouse to determine anti-sporozoite antibody levels and seven weeks later mice were challenged with non-irradiated *P. yoelii *sporozoites to determine liver stage protection.

**Figure 2:**
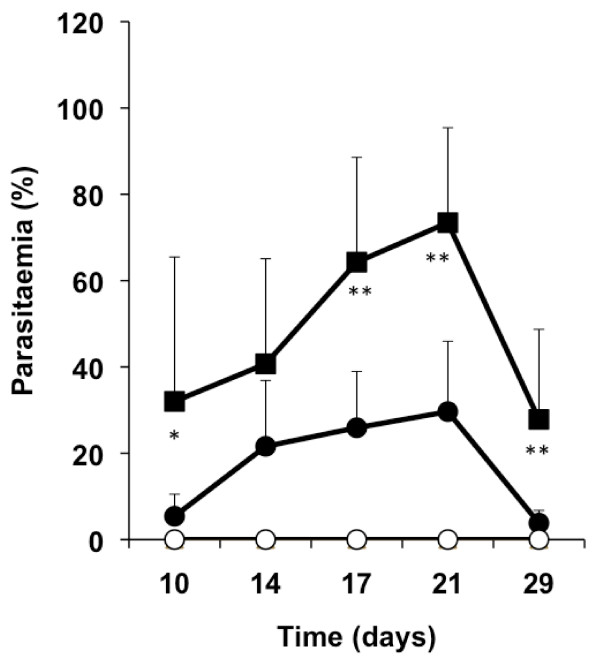
**Determination of parasitaemia**. Parasitaemia was determined in peripheral blood in all groups of mice every other day, starting ten days after the beginning of daily mosquito bites. Average parasitaemias are shown for each group. Parasitaemia in the group subjected to normal mosquito bites (black squares) and immunized followed by normal mosquito bites (black circles) are shown. In the groups subjected to irradiated mosquito bites (white circles) and immunized control (white squares) no infected erythrocytes were detected. Error bars represent standard deviation within groups of mice (*, *P *< 0.05; **, *P *< 0.01, when comparing mice subjected to normal mosquito bites and immunized followed by normal mosquito bites mice using ANOVA).

Parasitaemia was measured in each mouse weekly, confirming that irradiation of sporozoites and mosquitoes was effective; no blood stage infection was found in the group subjected to the bites of irradiated mosquitoes or the group immunized with irradiated sporozoites that did not receive mosquito bites. The data also show that mosquito bites induce a delayed development and lower levels of parasitaemia in mice that were previously immunized with irradiated sporozoites compared to non-immunized mice, probably reflecting the lower liver stage infection levels in the partially immunized mice (Figure 2).

When the levels of anti-sporozoite antibodies were determined, we observed that mice subjected to the bites of irradiated, infected mosquitoes develop significantly higher levels of antibodies compared to mice subjected to the bites of normal infected mosquitoes. It is important to note that both groups of mice had received equal numbers of mosquito bites and it is, therefore, expected that each group had received equal numbers of sporozoites.

**Figure 3:**
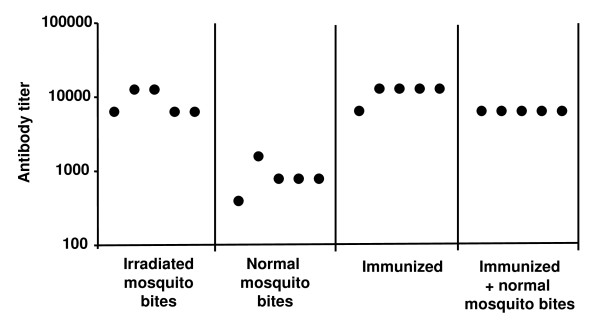
**Anti-sporozoite antibody titres**. Serum from each mouse was serially diluted and used to stain *P. yoelii *sporozoites. The titers represent the inverse of the highest dilution at which sporozoites could still be detected. Each circle represents an individual mouse. Significant differences between the group of normal mosquito bite and the other groups were found (*P *< 0.05) when comparing with ANOVA).

Antibody levels in the groups of mice that were immunized with two doses of the irradiated sporozoite vaccine followed or not by the bites of normal infected mosquitoes showed no significant differences in their anti-sporozoite antibody levels, suggesting that after immunization, the antibody levels are not affected by the bites of infected mosquitoes (Figure 3), even if the mice developed a full blood stage infection (Figure 2).

To study the level of immunization acquired by the different groups of mice, each mouse was challenged with non-irradiated *P. yoelii*-GFP sporozoites. It is important to use a *P. yoelii *strain that allows us to differentiate the challenge parasites from parasites (in liver or blood stage forms) that were inoculated before by either the immunization protocols or the mosquito bites. This is possible analysing the livers of challenged mice using real-time PCR to amplify the GFP sequence present only the challenge sporozoites. This technique is not as sensitive as the conventional RT-PCR used for detection of liver stage infection, which amplifies sequences within the 18S rRNA that are very abundant in the parasite [16].

Mice were challenged 14 weeks after being subjected to the first infected mosquito bite, when blood stage parasitaemia was undetectable. It was found that, while the liver stages of the GFP challenge sporozoites were not detectable in mice subjected to irradiated infected mosquito bites, mice that had been bitten by normal infected mosquitoes developed a detectable liver stage infection (Table 1). These results indicate a higher level of liver stage protection in mice subjected to irradiated infected mosquito bites. In this study the challenge was performed by i.v. injection of sporozoites, which is different from the natural environment, where mosquito bite inoculation would deposit sporozoites in the skin of the host. Despite these differences, the results observed in mice reflect the situation of humans, which are also protected by the bites of irradiated infected mosquitoes [5], but are not protected by natural exposure to infected mosquito bites [7]. It should be noted that this study was performed with two *P. yoelii*-infected mosquito bites per day, which is considered a high transmission rate and, therefore, it is not possible to extrapolate the results to lower frequencies of transmission, to a situation with concurrent bites of infected and uninfected mosquitoes or to infection with different strains of *Plasmodium*, which would reflect more accurately the conditions in malaria-endemic areas. In particular, the degree of variability of *Plasmodium *strains that is found in the field may interfere with the protection induced by irradiated infected mosquitoes.

**Table 1:** Protection against challenge

Mice experimental conditions	Threshold cycle (Ct) in real time-PCR	Number of mice (protected/total)
Irradiated mosquito bites	> 40	(3/3)
Normal mosquito bites	17 ± 0.85	(0/3)
Immunized	> 40	(3/3)
Immunized + normal mosquito bites	> 40	(3/3)

Groups of mice were challenged with 10^4 ^*P. yoelii*-GFP sporozoites injected intravenously. Real-time PCR was run for 40 cycles, therefore Ct > 40 indicates no detection of parasite in the liver.

In groups of mice that were immunized with irradiated sporozoites, subsequent infected mosquito bites, which caused a patent blood stage infection (Figure 2), did not induce a loss of the protection acquired with the experimental vaccination (Table 1). These results indicate that liver stage protection induced by irradiated sporozoites is not significantly affected by subsequent infection in mice. It is important to note that the groups of mice that were partially immunized with irradiated sporozoites probably developed a low-level liver stage infection. However, this level of infection is not detected by the GFP RT-PCR used in this assay.

Taken together, these data indicate that the generation of liver stage immunity against malaria is greatly dependent on whether the mosquitoes that transmit infection are irradiated or not. For both mice and humans, it has been extensively demonstrated that irradiated sporozoites induce a protective immune response [5,6], however, what remains unclear is why normal liver stage infection does not induce comparable protection. The possibility that low doses of sporozoites delivered in the skin by mosquito bites could induce tolerance to this stage of the parasite has been discarded in the mouse model [10]. It has been also proposed that the subsequent blood stage infection that follows the liver infection interferes with the generation of protection to the liver stage [12], a mechanism that could underlie the lack of protection observed in mice beaten by infective mosquitoes. It is possible that the high inflammatory immune response that blood stage infection generates could affect the establishment of a competent liver stage memory response that would be maturing at the same time as the blood stage is at its peak. It is also possible that the aborted liver infection that takes place with irradiated sporozoites generates a more protective immune response compared to liver infection by regular sporozoites. Previous experiments in mice suggest that this is not the case, because the liver stage response was found to be similar in mice infected with irradiated sporozoites compared to mice infected with normal sporozoites and treated to inhibit blood stage infection [12]. However, in this work [12] the response was only measured for CS protein and it is possible that the immune response to other proteins involved in *Plasmodium *liver stage protection [17] could be different when induced by irradiated versus regular sporozoites.

The analysis of mice that were first partially immunized with irradiated sporozoites and then subjected or not to infected mosquito bites indicates that liver stage protection was not lost after infective mosquito bites that result in blood stage infection. Since the mice were only partially protected, a full blood stage infection took place after mice were bitten by infective mosquitoes. This allowed us to analyze the effect of blood stage infection on the maintenance of protection induced by the vaccination. Contrarily to the immune suppression described when blood stage infection takes place right after the liver stage [12], the protection induced by the vaccination in our mice was not lost after blood stage infection that occurred four weeks after the first immunization (two weeks after the last immunization). These results suggest that after establishment of the protective liver stage immune response, this response is not significantly affected by subsequent blood stage infection.

## Conclusions

The results presented show that the liver stage immunity generated in response to irradiated versus normal *P. yoelii *infected mosquitoes is essentially different: while irradiated mosquitoes induce a protective immune response with high levels of anti-sporozoite antibodies, normal infected mosquitoes do not generate protection and only low levels of specific antibodies. This situation reflects the response of humans observed both in vaccination trials, where irradiated mosquito bites induce protection, and in the field, where years of constant re-infections do not result in sterile protection against the liver stage. The direct comparison of the human immune response to normal versus irradiated mosquito bites is not possible because the conditions of exposure to infective mosquito bites in the field are very different from experimental exposure to irradiated mosquito bites. In directly comparing the response generated by irradiated or non-irradiated infected mosquito bites, this work indicates that the mosquito irradiation is the only essential variable needed to generate protective liver stage response.

While infective mosquito bites do not induce a protective liver stage response, they also do not interfere with previously acquired liver stage protective responses, even if they induce a complete blood stage infection. Considering that the recently generated anti-malaria vaccines induce only partial protection against infection, it is encouraging that, at least in mouse models, immunity is not negatively affected by subsequent exposure and infection with the parasite.

## Competing interests

The authors declare that they have no competing interests.

## Authors' contributions

TP performed mice mosquito daily bite infections and measured parasitaemias. RL carried out mice immunizations, real-time PCR experiments and contributed to the writing of the manuscript. CGR carried out antibody quantifications. KAW conceived the study, participated in its design and trained TP. AR conceived the study, participated in its design and coordination and wrote the manuscript. All authors read and approved the final manuscript.
